# Molecular Profile of Advanced Radioiodine-refractory Thyroid Cancer and Response to Lenvatinib Treatment

**DOI:** 10.1210/clinem/dgag074

**Published:** 2026-03-27

**Authors:** Elisa Minaldi, Teresa Ramone, Raffaele Ciampi, Cristina Romei, Roberta Casalini, Carla Gambale, Alessandro Prete, Loredana Lorusso, Laura Agate, Liborio Torregrossa, Paolo Piaggi, Antonio Matrone, Rossella Elisei

**Affiliations:** Department of Clinical and Experimental Medicine, Endocrinology Unit, University of Pisa, Pisa 56124, Italy; Department of Clinical and Experimental Medicine, Endocrinology Unit, University of Pisa, Pisa 56124, Italy; Department of Clinical and Experimental Medicine, Endocrinology Unit, University of Pisa, Pisa 56124, Italy; Department of Clinical and Experimental Medicine, Endocrinology Unit, University of Pisa, Pisa 56124, Italy; Department of Clinical and Experimental Medicine, Endocrinology Unit, University of Pisa, Pisa 56124, Italy; Department of Clinical and Experimental Medicine, Endocrinology Unit, University of Pisa, Pisa 56124, Italy; Department of Clinical and Experimental Medicine, Endocrinology Unit, University of Pisa, Pisa 56124, Italy; Department of Clinical and Experimental Medicine, Endocrinology Unit, University of Pisa, Pisa 56124, Italy; Department of Clinical and Experimental Medicine, Endocrinology Unit, University of Pisa, Pisa 56124, Italy; Department of Surgical, Medical and Molecular Pathology, Pathology Unit, University of Pisa, Pisa 56124, Italy; Department of Information Engineering, University of Pisa, Pisa 56124, Italy; Department of Clinical and Experimental Medicine, Endocrinology Unit, University of Pisa, Pisa 56124, Italy; Department of Clinical and Experimental Medicine, Endocrinology Unit, University of Pisa, Pisa 56124, Italy

**Keywords:** thyroid carcinoma, radioiodine-refractory thyroid cancer, lenvatinib, BRAF, RAS

## Abstract

**Context:**

The predictive role of mutational status in the response to lenvatinib in advanced radioiodine-refractory thyroid cancer (RAIR-TC) is not yet defined.

**Objective:**

To identify a molecular signature of RAIR-TC treated with lenvatinib and its impact on clinical response to lenvatinib.

**Design:**

This is a retrospective study including 49 RAIR-TC patients treated with first-line lenvatinib and followed at least 1 year [median follow-up 9.2 years (interquartile range 6-14.1)]. Next-generation sequencing on tissues was used to detect genetic alterations.

**Setting:**

The study was performed in a referral center for the treatment of thyroid cancer.

**Results:**

Among 49 cases, *BRAF* (38.7%) and *RAS* (22.4%) were the most frequent driver mutations, while *TERT* and *TP53* were mutated in 57.1% and 6.1%. No driver mutations were found in 34.6%. Driver mutations co-occurred with *TERT* or *TP53* in 44.9%. In 10/49 (20.4%), no mutations were found. Cases with ≥1 mutation had longer progression-free survival (PFS) than cases negative for any mutation (*P* = .004). Both *BRAF*-mutated and *RAS*-mutated cases showed better PFS and overall survival (OS), although this was not statistically significant with respect to wild-type cases. Cases with a single driver mutation (group 1) showed longer PFS than cases without driver mutations (group 3) (*P* = .04) but no difference with cases with driver + *TERT* or *TP53* (group 2) (*P* = .13). Group 1 showed longer OS than group 2 (*P* = .026) and group 3 (*P* = .034).

**Conclusions:**

The RAIR-TC molecular profile is heterogeneous with *TERT* being the most frequent mutation. Cases negative for all mutations and cases presenting the coexistence of *TERT* + driver mutation had a worse response to lenvatinib. Conversely, the presence of 1 driver mutation correlated with a better response to lenvatinib.

Many clinical factors have been described as potential influencers of response to multikinase inhibitors therapy in patients with advanced radioiodine-refractory thyroid carcinoma (RAIR-TC), such as older age, male sex, histological subtype, presence of liver or brain metastasis, higher thyroglobulin levels, baseline tumor size, tumor volume doubling time, larger size of lung metastasis, and neutrophil-to-lymphocyte ratio (1-9).

The pathogenesis of thyroid cancer (TC) is characterized by mutations mainly affecting the MAPK and *PI3K*/*AKT* pathways (10). The most frequent driver alterations can be either point mutations or gene rearrangements and are usually mutually exclusive (11). The mutated genes, which are considered the drivers of tumoral transformation, encode cell-membrane receptors with tyrosine-kinase activity, such as *RET* and *NTRK*, and intracellular signal transducers, including *BRAF* and *RAS*. Other alterations, such as *TP53* and *TERT* promoter mutations, are likely involved in TC progression (12), and their association with one of the drivers is considered a factor for poor prognosis (13, 14).

Limited data are available regarding the impact of the mutational status of RAIR-TC on the clinical response to multikinase inhibitors. A subanalysis of the sorafenib phase III DECISION trial showed that *BRAF* and *RAS* mutations were associated with better and worse progression-free survival (PFS), respectively (5). However, these factors lose significance in a multivariate analysis that includes clinical variables. Differently from what was observed with sorafenib, an exploratory analysis from the SELECT study cohort reported no significant differences in clinical outcomes in *BRAF* or *RAS* mutated cases treated with lenvatinib (6).

The present study aimed at identifying a common molecular profile in RAIR-TC and assessing its impact on clinical response to lenvatinib in a series of RAIR-TC treated and followed up at a single referral center.

## Patients and Methods

### Patients’ Cohort

We evaluated 198 patients with both differentiated thyroid cancer (DTC) and poorly differentiated thyroid cancer (PDTC) treated with lenvatinib and followed at the Endocrine Unit of the University Hospital of Pisa, Italy, in the period 2012-2023. The inclusion criteria for the study were as follows: (1) surgical treatment performed at the University Hospital of Pisa; (2) positive histology for DTC or PDTC, with the latter according to Turin criteria (15); (3) formalin-fixed paraffin-embedded tissues of primary and/or lymph nodes and/or distant metastases available and archived at the same institution; (4) patients treated with lenvatinib as first-line systemic therapy; (5) last follow-up visit at least 1 year after lenvatinib start or deceased before.

**Figure 1:**
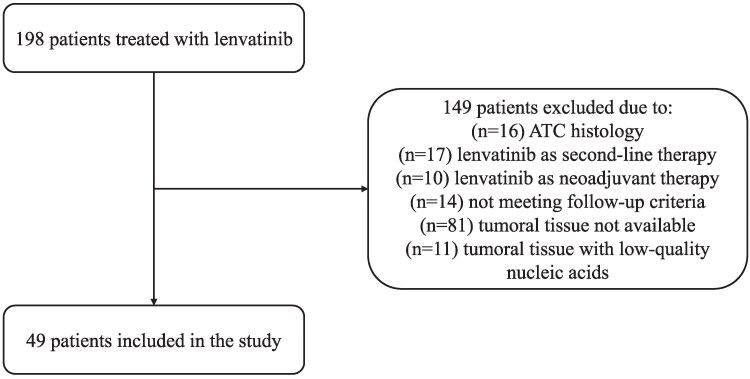
Patient selection flowchart: patients were selected according to inclusion criteria. Cases operated in other institutions were excluded because of the unavailability of the tumoral tissue.

After this strict selection, which was necessary to avoid any possible bias, a total of 49 patients were enrolled (Fig. 1). Regarding available tissues, 25/49 (51%) were primary tumors, 21/49 (42.9%) were lymphnode metastases, and 3/49 (6.1%) were distant metastases.

Patients received lenvatinib through either participation in clinical trials or postapproval commercial treatment. The clinical trials for lenvatinib included the SELECT study (NCT01321554) (3) and the expanded access program study (16).

Patients signed several consent forms both to participate in clinical trials in which they were treated with the drug and to participate in this study. The study was approved by the local ethical committee EAVNO: Comitato Etico Area Vasta Nord Ovest; Protocol number: 2017YTWKWH).

### Follow-up Strategy

Patient data were collected before starting lenvatinib treatment and during follow-up. The initial dose for all patients in the SELECT and expanded access program studies was 24 mg daily. For compassionate use or postapproval cases, the drug was started at 24 mg/day or lower based on the patient's condition (either 20, 14, 10, or 8 mg per day). Dose adjustments (in case of G1-G2 adverse events) or temporary interruptions (in case of G3-G4 adverse events) occurred if severe adverse events, according to the Common Terminology Criteria for Adverse Events, emerged (17).

All patients underwent an assessment at baseline and, subsequently, according to the clinical trial schedule; the follow-up intervals were similar in all patients receiving lenvatinib. Patients were examined every 2 months during the first year and then every 6 months. At each control, patients were submitted to neck ultrasound, clinical examination, and laboratory assessment (thyroglobulin and thyroglobulin autoantibody, thyroid function, routine exams) and dual-phase total-body computed tomography scan with IV contrast medium. Clinical response to lenvatinib was assessed by computed tomography scans according to Response Evaluation Criteria in Solid Tumours (RECIST1.1) (18). Additional imaging techniques, such as magnetic resonance imaging, positron emission tomography with 18-fluorodeoxyglucose, and bone scans, were performed if medical judgment considered them essential.

### Nucleic Acid Isolation From Tumoral Tissues

DNA and RNA were extracted from formalin-fixed paraffin-embedded tissues. The tumoral area and tumoral cellularity were identified by our pathologist (L.T.). A commercial assay, AllPrep DNA/RNA kit (Qiagen), was used to obtain genomic DNA and total RNA following the manufacturer's instructions. The Qubit dsDNA and RNA High Sensitivity Assay Kit was used to measure concentration by using a Qubit fluorometer (Thermo Fisher).

### Next-generation Sequencing Data Analysis

#### Next-generation sequencing of DNA

Point mutations and small indels of the most relevant oncogenes involved in thyroid tumorigenesis were analyzed using our custom thyroid-specific panel, whose characteristics have been previously reported (19). For each sample, 10 ng of DNA obtained from tumoral tissue was used to prepare libraries using the Ion AmpliSeq™ Kit for Chef DL8 (Thermo Fisher) on the Ion Chef System (Thermo Fisher Scientific, Carlsbad, CA). Libraries were pooled together, and the Ion 510™, Ion 520™, and Ion 530™ Kit–Chef (Thermo Fisher) were used in the Ion Chef System for template preparation. Deep sequencing was performed on an Ion S5 system (Thermo Fisher). Raw data were automatically processed on the Torrent Server™ and aligned to the reference hg19 genome. Further analyses were performed using the Ion Reporter™ Analysis Server (Thermo Fisher Scientific) for variant calling and annotation.

#### Next-generation sequencing of RNA

Cases that were negative for point mutations or small insertion-deletions were tested for the presence of gene fusions. For gene fusions analysis, we used another custom panel containing 40 specific gene fusions involved in thyroid carcinoma tumorigenesis affecting *RET*, *BRAF*, *NTRK1*, *NTRK3*, *ALK*, *ROS1*, and *PPARγ* genes; the panel also included assays for the 5′-3′ imbalance of *RET, BRAF, NTRK1*, *NTRK3*, *ALK*, and *ROS1* to detect the presence of hypothetical novel fusions alternative to the already known fusions. Sequencing was performed using 12 ng of total RNA, which were reverse-transcribed in cDNA using Superscript IV Vilo (Thermo Fisher Scientific Inc., Waltham, MA); libraries were prepared similarly to what has been described for DNA following the manufacturer's instructions. The oncoprint was designed using the cBioPortal tool (20, 21).

### *TERT* Promoter Mutation Analysis

*TERT* promoter mutations were separately analyzed by droplet digital PCR. In particular, C228T and C250T mutations were analyzed using the specific ddPCRBioRad assays dHsaEXD72405942 and dHsaEXD46675715, respectively. The reaction mix was prepared according to the manufacturer's instructions.

### Statistical Analysis

Data were expressed as mean ± SD or median with interquartile range and minimum to maximum, where appropriate. Normality was tested using the Shapiro-Wilk test. Categorical variables were compared by the χ^2^ test, while continuous variables were compared using Student's *t*-test, based on variance assessment by the Levene test. Nonparametric continuous variables were compared using the Mann–Whitney U-test. The Kaplan-Meier method was used to estimate overall survival (OS) and PFS. Log-rank or the Breslow test was used to assess differences between groups in OS or PFS. Cox proportional hazard model analysis was used to estimate the cumulative risk (hazard) of death or disease progression and to determine the independent effect of the mutational profile on these outcomes. The statistical significance level was set at *P* < .05. Data analysis was performed using IBM SPSS Statistics software.

## Results

### Clinical and Pathological Features

Epidemiologic, clinical, and pathological features of the study group are shown in Table 1. Males and females were equally distributed, and the median age at diagnosis was 60.5 years [interquartile range (IQR) 50.7-68.4; range 40.5-85.4]. Most of the cases were papillary thyroid cancer (PTC) (51%; n = 25) or follicular thyroid cancer (FTC) (28.6%; n = 14), while each 1 of the PDTC and oncocytic thyroid carcinoma (OTC) histotypes accounted for 10.2% (n = 5) of the whole group. Distant metastases at diagnosis were detected in 30.6% (n = 15) of the cases, mainly in the lungs (66.7%; n = 10), followed by the bone (13.3%; n = 2), while in 3 cases (20%), both sites were involved. American Joint Committee on Cancer staging at diagnosis was distributed as follows: 16 (32.7%) cases were classified as stage 1, 15 (30.6%) as stage 2, 6 (12.2%) as stage 3, and 12 (24.5%) as stage 4B. When we arbitrarily calculated the initial stage without considering the age at diagnosis, the staging distribution was as follows: 3 (6.1%) cases were classified as stage 1, 22 (44.9%) as stage 2, 8 (16.3%) as stage 3, 1 (2%) as stage 4A, and 15 (30.6%) as stage 4B.

**Table 1:** Epidemiological, clinical, and pathological features of a cohort of patients with advanced thyroid cancer treated with lenvatinib

	Patients n = 49
Females (n (%))	26 (53.1)
Males (n (%))	23 (46.9)
Age at diagnosis (years)	Median 60.54 (IQR 50.7-68.4)
Age < 55 years (n (%))	19 (38.8)
Age > 55 years (n (%))	30 (61.2)
Histotype (n (%))	
PTC	25 (51)
FTC	14 (28.6)
PDTC	5 (10.2)
OTC	5 (10.2)
T1a	1 (2)
T1b	—
T2	14 (28.6)
T3a	9 (18.4)
T3b	10 (20.4)
T4a	11 (22.4)
T4b	2 (4.1)
Tx	2 (4.1)
N0	18 (36.7)
N1a	3 (6.1)
N1b	25 (51)
Nx	3 (6.1)
M0	34 (69.4)
M1	15 (30.6)
Lung	10 (66.7)
Bone	2 (13.3)
Lung + bone	3 (20)
Stage (n (%))	
1	16 (32.7)
2	15 (30.6)
3	6 (12.2)
4A	—
4B	12 (24.5)
Stage without considering age (n (%))	
1	3 (6.1)
2	22 (44.9)
3	8 (16.3)
4A	1 (2)
4B	15 (30.6)

Abbreviations: FTC, follicular thyroid cancer; IQR, interquartile range; OTC, oncocytic thyroid cancer; PDTC, poorly differentiated thyroid cancer; PTC, papillary thyroid cancer.

Patients were followed for a median time of 9.2 years (IQR 6-14.1; range 0.6-21.4). The median age at the time of lenvatinib start was 69.4 years (IQR 59.7-75; range 43-89.1), with a median time from the diagnosis to the lenvatinib start of 88.9 months (IQR 34.1-146.4; range 1.4-243.8), and a median time from the radioiodine-refractoriness diagnosis of 29.7 months (IQR 14.8-57.3; range 3.7-153.9). The median duration of lenvatinib therapy was 23.1 months (IQR 8-38.5; range 1-98.4).

Overall, 35/49 (71.4%) patients died: 27/35 (77.1%) due to progression or adverse events related to their TC, 5/35 (14.3%) due to lenvatinib-related adverse events (2 for hemorrhagic stroke, 3 for hemorrhage secondary to tracheal perforation), and 3/35 (8.6%) due to other causes (2 for pneumonia and 1 for cholangiocarcinoma). Considering the deceased patients only, median OS since diagnosis was 9.9 years [95% confidence interval (CI) 7.4-12.4] and median OS since lenvatinib start was 20.7 months (95% CI 7.6-33.8).

Considering the whole cohort, the median OS from initial diagnosis was 11.7 years (95% CI 8.3-15.2), while the median OS after lenvatinib start was 36.5 months (95% CI 23.1-49.9). The median PFS was 18 months (95% CI 12.1-23.8).

Five out of 49 (10.2%) patients underwent second-line systemic therapy after lenvatinib: 4/5 with cabozantinib and 1/5 with the combination dabrafenib-trametinib. It is noteworthy that in these cases, the follow-up was censored at the end of the first-line therapy with lenvatinib.

### Mutational Profile

**Figure 2:**
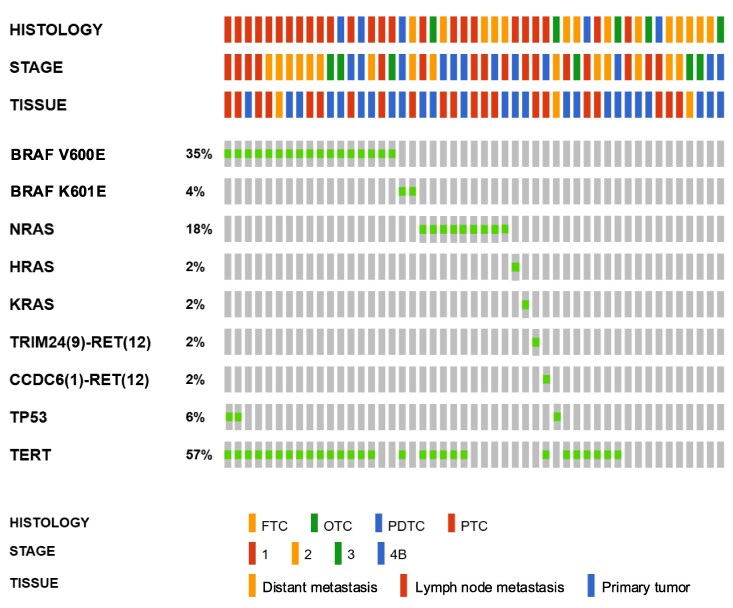
Oncoprint showing the molecular alterations in 49 patients with advanced radioiodine-refractory differentiated and poorly differentiated thyroid cancer treated with lenvatinib.

The type and the distribution of molecular alterations found in our series are reported in Fig. 2. Molecular alterations, 1 or more than 1, were detected in most cases (n = 39/49; 79.6%), while in the remaining 10/49 (20.4%) patients no mutations already known to be involved in thyroid tumorigenesis according to our custom panel were identified. Among all patients, 10/49 (20.4%) cases harbored a single driver mutation that was *BRAF*V600E (n = 2; 4.1%), *BRAF*K601E (n = 1, 2.1%), *NRAS* (n = 4; 8.2%), *HRAS* (n = 1; 2%), *KRAS* (n = 1; 2%), and *CCDC6(1)-RET(12)* (n = 1; 2%). In 22/49 (44.9%) cases, a driver mutation coexisted with an additional mutation (*TERT* or *TP53*). Seven cases (14.3%) were negative for driver mutations but carried *TERT* alone in 6/49 (12.2%) cases and *TP53* alone in 1/49 (2%) cases. *TERT-*mutated cases (n = 26; 57.1%) harbored *TERT*C228T mutation in most cases (n = 26; 92.9%), while *TERT*C250T was present in 2 cases (n = 2; 7.1%).

Moreover, beyond the previously reported somatic mutations, we identified mutations in the *EIF1AX, CHEK2, AKT1, STK11, PTEN*, and *TP53* genes, either alone or in combination with another driver mutation. Since they were all classified as either variants of uncertain significance or benign/likely benign according to the ClinVar database (https://www.ncbi.nlm.nih.gov/clinvar/), they were not included in the correlation with clinical outcome.

### Molecular Profile and Response to Therapy

**Figure 3:**
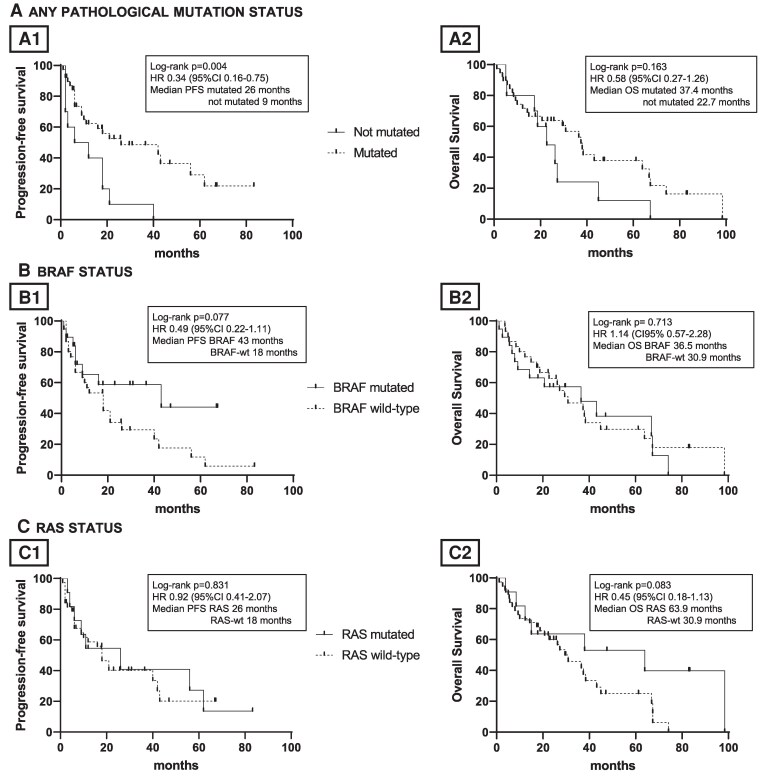
Kaplan-Meier estimates of progression-free survival and overall survival since lenvatinib start in patients with advanced differentiated or poorly differentiated thyroid cancer treated with lenvatinib stratified for mutational status: (A) ≥1 vs no mutations; (B) *BRAF* status: *BRAF*-mutated vs *BRAF-*wt; (C) *RAS* status: *RAS*-mutated vs *RAS*-wt. Abbreviation: wt, wild-type.

To analyze the association between molecular impact and clinical outcomes, we first distinguished 2 groups according to the presence or absence of any mutation. The clinical and pathological features of patients belonging to the 2 groups are reported in Table 2: no major differences were observed except for a statistically significant greater number of PTC in the mutated group. When we analyzed the PFS and the OS of the 2 groups, we observed that mutated cases had a statistically significant 17 months longer PFS than not-mutated cases [log-rank test *P* = .004; hazard ratio (HR) 0.34; 95% CI 0.16-0.75; *P* = .007] (Fig. 3, panel A1). Also, the OS of mutated cases was about 15 months longer than that of nonmutated, but this difference did not reach statistical significance (log-rank test *P* = .16; HR 0.58; 95% CI 0.27-1.26) (Fig. 3, panel A2). Due to the significant predominance of PTC in mutated group, to verify if the longer PFS was actually determined by histology, we performed a univariate analysis assessing the relationship between PFS and OS and histology (PTC vs not PTC). Both PFS and OS were not significantly associated with the histology of PTC (PFS: HR 0.61, 95% CI 0.3-1.25, *P* = .18; OS: HR 1.21, 95% CI 0.61-2.39, *P* = .58).

**Table 2:** Epidemiological, clinical, and pathological features of 49 patients with advanced thyroid cancer treated with lenvatinib divided into 2 groups according to presence or absence of any pathological mutation

	Mutated (n = 39)	Not mutated (n = 10)	*P*
Females (n (%))	22 (56.4)	4 (40)	.48
Males (n (%))	17 (43.6)	6 (60)	
< 55 years old (n (%))	13 (33.3)	4 (40)	.72
> 55 years old (n (%))	26 (66.7)	6 (60)	
Histotype (n (%))			**.022**
PTC	24 (61.5)	1 (10)	
FTC	8 (20.5)	6 (60)	
PDTC	4 (10.3)	1 (10)	
OTC	3 (7.7)	2 (20)	
T1a	—	1 (10)	.29
T1b	—	—	
T2	13 (33.3)	1 (10)	
T3a	7 (17.9)	2 (20)	
T3b	8 (20.5)	2 (20)	
T4a	8 (20.5)	3 (30)	
T4b	1 (2.7)	1 (10)	
Tx	2 (5.1)	—	
N0	11 (28.2)	7 (70)	.06
N1a	3 (7.7)	—	
N1b	23 (59)	2 (20)	
Nx	2 (5.1)	1 (10)	
M0	27 (69.2)	7 (70)	.96
M1	12 (30.8)	3 (30)	
Stage (n (%))			.86
1	13 (33.3)	3 (30)	
2	12 (30.8)	3 (30)	
3	4 (10.3)	2 (20)	
4A	—	—	
4B	10 (25.6)	2 (20)	

Statistically significant values are reported in bold.

Abbreviations: FTC, follicular thyroid cancer; OTC, oncocytic thyroid cancer; PDTC, poorly differentiated thyroid cancer; PTC, papillary thyroid cancer.

Then we distinguished the cohort according to the presence or absence of *BRAF* mutation (*BRAF*V600E and *BRAF*K601E) and the presence or absence of *RAS* mutations (*H-K-NRAS*): *BRAF*-negative cases included all the other cases with or without any other mutation; similarly, *RAS-*negative cases included all the other cases with or without any other mutation. While the groups *RAS*-positive and *RAS*-negative were similar for all the clinical and pathological features analyzed (Table 3), the *BRAF*-mutated cases showed a statistically significant greater number of PTC with respect to *BRAF*-negative cases (Table 4). As shown in Fig. 3B, the PFS (panel B1) and the OS (panel B2) of *BRAF*-mutated cases were 25 and 5 months longer than that of *BRAF*-negative cases, respectively, but these differences were not statistically significant. For *RAS*-positive cases (Fig. 3C), the PFS (panel C1) and the OS (panel C2) were 8 months and 33 months longer than that of *RAS-*negative cases but, again, no statistically significant differences were found.

**Table 3:** Epidemiological, clinical, and pathological features of 49 patients with advanced thyroid cancer treated with lenvatinib divided into 2 groups according to presence or absence of *RAS* mutation

	*RAS* mutated (n = 11)	*RAS* wild-type (n = 38)	*P*
Females (n (%))	6 (54.5)	20 (52.6)	.91
Males (n (%))	5 (45.5)	18 (47.4)	
< 55 years old (n (%))	4 (36.4)	13 (34.2)	.89
> 55 years old (n (%))	7 (63.6)	25 (65.8)	
Histotype (n (%))			.61
PTC	6 (54.5)	19 (50)	
FTC	4 (36.4)	10 (26.3)	
PDTC	—	5 (13.2)	
OTC	1 (9.1)	4 (10.5)	
T1a	—	1 (3.3)	.59
T1b	—	—	
T2	4 (36.4)	7 (23.3)	
T3a	3 (27.3)	6 (20)	
T3b	2 (18.2)	5 (16.7)	
T4a	—	7 (23.3)	
T4b	1 (9.1)	2 (6.7)	
Tx	1 (9.1)	2 (6.7)	
N0	6 (54.5)	12 (31.6)	.36
N1a	1 (9.1)	2 (5.3)	
N1b	3 (27.3)	22 (57.9)	
Nx	1 (9.1)	2 (5.3)	
M0	6 (54.5)	28 (73.7)	.275
M1	5 (45.5)	10 (26.3)	
Stage (n (%))			.68
1	5 (45.5)	11 (28.9)	
2	1 (9)	14 (36.8)	
3	—	6 (15.8)	
4A	—	—	
4B	5 (45.5)	7 (18.5)	

Abbreviations: FTC, follicular thyroid cancer; OTC, oncocytic thyroid cancer; PDTC, poorly differentiated thyroid cancer; PTC, papillary thyroid cancer.

**Table 4:** Epidemiological, clinical, and pathological features of 49 patients with advanced thyroid cancer treated with lenvatinib divided into 2 groups according to presence or absence of *BRAF* mutation

	*BRAF* mutated (n = 19)	*BRAF* wild-type (n = 30)	*P*
Females (n (%))	10 (52.6)	16 (53.3)	.96
Males (n (%))	9 (47.4)	14 (46.7)	
< 55 years old (n (%))	5 (26.3)	12 (40)	.33
> 55 years old (n (%))	14 (73.7)	18 (60)	
Histotype (n (%))			**.002**
PTC	15 (78.9)	10 (33.3)	
FTC	1 (5.3)	13 (43.3)	
PDTC	3 (15.8)	2 (6.7)	
OTC	—	5 (16.7)	
T1a	—	1 (3.3)	.59
T1b	—	—	
T2	7 (36.8)	7 (23.3)	
T3a	3 (15.8)	6 (20)	
T3b	5 (26.3)	5 (16.7)	
T4a	4 (21.1)	7 (23.3)	
T4b	—	2 (6.7)	
Tx	—	2 (6.7)	
N0	2 (10.5)	16 (53.3)	**.002**
N1a	1 (5.3)	2 (6.7)	
N1b	16 (84.2)	9 (30)	
Nx	—	3 (10)	
M0	16 (84.2)	18 (60)	.073
M1	3 (15.8)	12 (40)	
Stage (n (%))			.39
1	5 (26.3)	11 (36.7)	
2	8 (42.1)	7 (23.3)	
3	3 (15.8)	3 (10)	
4A	—	—	
4B	3 (15.8)	9 (30)	

Statistically significant values are reported in bold.

Abbreviations: FTC, follicular thyroid cancer; OTC, oncocytic thyroid cancer; PDTC, poorly differentiated thyroid cancer; PTC, papillary thyroid cancer.

**Figure 4:**
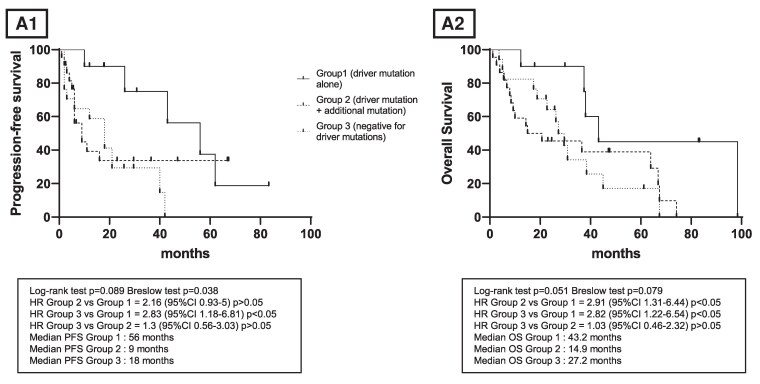
Kaplan-Meier estimates of progression-free survival and overall survival of patients with advanced differentiated or poorly differentiated thyroid cancer treated with lenvatinib stratified for mutational status: group 1: presence of driver mutation alone (*BRAF*V600E*, BRAF*K601E*, H-K-NRAS, RET/PTC* fusions); group 2: presence of driver mutation with an additional mutation (*TERT* and/or *TP53*); group 3: negative for driver mutations with or without additional mutations.

In order to evaluate the effect of the association of more than 1 mutation on the outcome, we distinguished the cohort in 3 groups according to the presence of 1 single driver mutation (either *BRAF*V600E *or BRAF*K601E or *H-K-NRAS* or *RET*/*PTC* fusions) (group 1), the presence of the driver with a second mutation (either *TERT* or *TP53*) (group 2), and the absence of driver mutations with or without additional mutations (*TERT* or *TP53*) (group 3). Some statistically significant differences were observed when looking at the clinical and pathological features, in particular the histotype distribution, with the complete absence of PDTC and OTC in group 1 and a higher prevalence of N1b in group 2 (Table 5). Note that in group 1, although not statistically significant, there was a higher percentage of females, younger patients, and smaller tumors; a lower percentage of metastatic disease at diagnosis; and a greater number of stage 1 cases. As shown in Fig. 4, panel A, group 1 had a statistically significant much longer PFS with respect to the other groups (47 months longer than group 2; 38 months longer than group 3). A similar trend was observed for the OS (Fig. 4, panel B), which was 28.3 and 16 months longer in group 1 with respect to groups 2 and 3, respectively, but in this case the difference was not statistically significant.

**Table 5:** Epidemiological, clinical, and pathological features of 49 patients with advanced thyroid cancer treated with lenvatinib grouped based on molecular profile

	Group 1	Group 2	Group 3	*P*
	Presence of driver mutation	Driver + additional mutation	No driver mutations	
Females (n (%))	6 (60)	12 (54.5)	8 (47)	.79
Males (n (%))	4 (40)	10 (45.5)	9 (53)	
< 55 years old (n (%))	6 (60)	6 (27.3)	7 (41.2)	.61
> 55 years old (n (%))	4 (40)	16 (72.7)	10 (58.8)	
Histotype (n (%))				**.001**
PTC	6 (60)	17 (77.3)	2 (11.8)	
FTC	4 (40)	1 (4.5)	9 (52.9)	
PDTC	—	3 (13.7)	2 (11.8)	
OTC	—	1 (4.5)	4 (23.5)	
T1a	—	—	1 (5.9)	.83
T1b	—	—	—	
T2	4 (40)	7 (31.9)	3 (17.6)	
T3a	2 (20)	4 (18.2)	3 (17.6)	
T3b	2 (20)	6 (27.3)	2 (11.8)	
T4a	2 (20)	3 (13.6)	6 (35.3)	
T4b	—	1 (4.5)	1 (5.9)	
Tx	—	1 (4.5)	1 (5.9)	
N0	5 (50)	3 (13.6)	10 (58.8)	**.04**
N1a	—	2 (9.1)	1 (5.9)	
N1b	5 (50)	16 (72.8)	4 (23.5)	
Nx	—	1 (4.5)	2 (11.8)	
M0	8 (80)	15 (68.2)	11 (64.7)	.69
M1	2 (20)	7 (31.8)	6 (35.3)	
Stage (n (%))				.39
1	6 (60)	5 (22.7)	5 (29.4)	
2	1 (10)	8 (36.3)	6 (35.4)	
3	1 (10)	2 (9.1)	3 (17.6)	
4A	—	—	—	
4B	2 (20)	7 (31.8)	3 (17.6)	

Group 1: presence of driver mutation alone (either *BRAF* or *RAS* or *RET/PTC*); group 2: presence of driver mutation with additional mutation (*TERT* and/or *TP53*); group 3: negative for driver mutations with or without additional mutations. Statistically significant values are reported in bold.

## Discussion

In this paper, we analyzed the molecular profile of a cohort of patients with advanced RAIR-TC treated with lenvatinib. Regarding the first aim of the study, we were unable to find a “common” molecular profile for RAIR-TC. We confirmed that *BRAF* mutations, taken together *BRAF*V600E and *BRAF*K601E, were the most frequent genetic alterations (39%), followed by *RAS* mutations (22%). However, this finding is likely due to the higher number of PTC compared to FTC, in which the *BRAF*V600E mutation is generally very frequent or null, respectively.

Note that 34.6% cases of the whole study group did not harbor any driver mutation, and most of these negative cases were OCT or FTC. The negativity of these cases can only be defined for the genes covered by our custom next-generation sequencing panel, which are all those that, so far, have been recognized as relevant for the thyroid tumorigenesis (19). We could hypothesize that other unknown mutations or somatic copy number alterations or epigenetic modifications may act as drivers (11, 22).

Among PTCs, *BRAF* mutations were present in 68% of cases. This frequency is similar to that found in previous studies that analyzed the genetic landscape of advanced cases of PTC (23-25). We found that *TERT* promoter mutation was the second most frequent alteration (57% of the whole cohort and 72% of all PTC), similar to other cohorts of advanced PTC (24, 25), but the frequency was markedly higher than that reported by The Cancer Genome Atlas (11). One possible explanation for this difference can be related to the selection of cases, since the PTC of our cohort were all advanced and RAIR, while in The Cancer Genome Atlas the cases were all well-differentiated and included all risk categories.

Moreover, in 2 out of 10 (20%) PTCs that were negative for *BRAF* and *RAS* mutations, we identified *RET* rearrangements, specifically *CCDC6(1)-RET(12)* and *TRIM24(9)-RET(12).* The prevalence of *RET* rearrangements in our cohort aligns with findings from a Canadian study (26), which reported *RET* fusions in 4 out of 20 *BRAF*-negative cases (20%) of RAIR-TC with distant metastases.

Among FTCs of our cohort, 4/14 (28.6%) harbored *RAS* mutations. This frequency is different from the one reported in the study by Pozdeyev et al (66%), which included 65 cases of advanced FTC (24). Probably, the group of FTC in our study is too small to make any comparison and draw formal conclusions.

We found a very low prevalence of *TP53* mutations, but it is worth recalling that in this series there are no anaplastic TCs, which are the TC histotype characterized by the copresence of *TP53* mutations with other drivers (27, 28).

The small group of PDTC in our study had a quite heterogeneous molecular profile, including 3 cases with the association of *BRAF* (2 cases *BRAF*V600E and 1 case *BRAF*K601E) and *TERT* promoter mutation, with 1 case harboring a *TERT* mutation alone and 1 negative for any mutation searched. Despite the small number of cases, this result confirms other reports that found *TERT* promoter mutations to be the most common alteration in PDTC (27, 28).

Regarding the few cases of OTC in the present study, we found that only 1 case harbored a *N-RAS* mutation, while 4/5 cases were negative for drivers. Moreover, *TERT* promoter was mutated in 3/5 cases and *TP53* in 1/5 cases. This partially reflects the results of the study by Gainly et al on 56 cases of Hürthle cell TC (now reclassified as OTC), in which driver mutations were very rare, with *NRAS* being mutated in 9% of cases and other mutations such as *TERT* in 22% and *TP53* in 7% of cases (29).

Regarding the second aim of the study, we found a positive correlation between the presence of any mutation and a longer PFS under lenvatinib, and, in particular, we observed that cases harboring a driver mutation (either *BRAF*V600E or *BRAF*K601E or *H-K-NRAS* or *RET*/*PTC* fusions) had a better response to lenvatinib, both for PFS and OS. Our results are only partially in line with the results of the post hoc SELECT study analysis (6), which showed a lenvatinib benefit on PFS for all subgroups regardless of tumor *BRAF* or *RAS* mutational status. This apparent difference could be due to the fact that they separated the groups according to the presence/absence of either *BRAF* or *RAS* mutations while we performed our first analysis putting together all mutated cases. However, when we analyzed our cases by separating *BRAF* and *RAS* mutated cases, we had similar results to Tahara et al (6). These authors found that *BRAF* mutation was not predictive of lenvatinib response, but it was identified as a prognostic factor for better PFS in PTC cases, similarly to our findings. Conversely, in the Tahara et al study, *RAS* mutations were neither predictive of lenvatinib response nor prognostic of PFS (6), in line with our results regarding *RAS* mutations.

One hypothesis to explain the finding that, during lenvatinib treatment, positive cases for any mutation had a better prognosis in terms of PFS compared to negative cases, could be that negative cases might have a worse prognosis independently from lenvatinib treatment. To our knowledge, there are no prospective studies analyzing the biological behavior of mutated and not-mutated and untreated advanced TC. However, in the study by Tahara et al, looking at the behavior of mutated and not-mutated cases treated with placebo, it appears that not-mutated cases had, per se, a slightly worse PFS with respect to mutated cases, thus supporting the hypothesis that not-mutated cases have a more aggressive biological behavior (6).

Another hypothesis to explain our results might be that lenvatinib could have better efficacy on cancer cells harboring a driver mutation. It is known that in tumors harboring a driver mutation, the MAP kinase signaling pathway is hyperactivated, and this activation is even reinforced by the activities of some tyrosine kinase receptors, such as vascular endothelial growth factor receptors and epidermal growth factor receptors (30, 31). The high anti-vascular endothelial growth factor receptor activity of lenvatinib can lead to the inhibition of these intracellular pathways, contributing to the blocking of the cellular growth (32). Conversely, in tumor cells that do not harbor driver mutations activating the MAP kinase pathway, other mechanisms of aggressiveness may be involved, against which lenvatinib could be less effective.

*TERT* promoter mutations have been shown to interact with *BRAF* mutations, and the coexistence of *BRAF* and *TERT* promoter mutations is a factor associated with poor prognosis in PTC (13, 14). In our study, the presence of these additional mutations to the driver seems to play a negative role on OS but not on PFS. Also, we found that the presence of additional mutations of worse prognosis (ie, *TP53* and *TERT*) determined a worse OS independently from the histotype of our cases.

Our results are apparently in contrast with a recent large study involving 552 advanced TCs, including medullary and anaplastic TC, requiring systemic therapy. The authors did not find a significant difference between patients with and without these coexisting mutations (23). However, the Japanese study includes a mix of different histotypes and drugs, which could represent confounding factors, as well as the inclusion of both treated and untreated patients. Our much smaller study, in terms of the number of enrolled patients, is more focused on the impact of genetic alterations in TC derived from follicular cells, with all patients treated with lenvatinib. These more restrictive enrollment criteria could explain the difference between our results and those of our Japanese colleagues.

One limitation of our study is that, in some sporadic cases, the tumoral tissue analyzed originated from lymphnode or distant metastasis. We can suppose that metastatic tissues might harbor additional mutations such as *TERT, TP53, PIK3CA,* and *AKT1,* differently from the primary tumor. Although we acknowledge that this could influence the results, there is a robust literature confirming the concordance between the presence of driver mutations in the primary tumor and their metastasis (33, 34), thus belittling the limitation itself.

While the heterogeneity and the relatively small sample size of our cohort may represent another limitation, at variance, in our study they may represent a strength when considering the rarity of RAIR-TC and the fact that they were collected and treated at a single center, followed up for a rather long time, and extensively analyzed by next-generation sequencing.

In conclusion, our study highlights the complex and highly variable genetic landscape of advanced RAIR-TC, varying from cases with multiple mutations and cases totally negative. We found that *BRAF* and *TERT* promoter mutations were prevalent in this cohort, particularly in PTC, with their coexistence correlating with poor prognosis and poor response to lenvatinib. Similarly, we observed that cases negative for all mutations were poor responders to the drug. Conversely, the presence of mutations in general and of drivers in particular was correlated with a better response to lenvatinib.

## Data Availability

Original data generated and analyzed during this study are included in this published article or in the data repositories listed in References.
